# Shorter fixation durations for up-directed saccades during saccadic exploration: A meta-analysis

**DOI:** 10.16910/jemr.12.8.5

**Published:** 2020-03-01

**Authors:** Harold H Greene, James M Brown, Gregory P Strauss

**Affiliations:** University of Detroit Mercy, USA; University of Georgia, Athens, USA

**Keywords:** Eye movement, saccades, fixation duration, saccade direction, meta-analysis

## Abstract

Utilizing 23 datasets, we report a meta-analysis of an asymmetry in presaccadic fixation durations for saccades directed above and below eye fixation during saccadic exploration. For inclusion in the meta-analysis, saccadic exploration of complex visual displays had to have been made without gaze-contingent manipulations. Effect sizes for the asymmetry were quantified as Hedge’s g. Pooled effect sizes indicated significant asymmetries such that during saccadic exploration in a variety of tasks, presaccadic fixation durations for saccades directed into the upper visual field were reliably shorter than presaccadic fixation durations for saccades into the lower visual field. It is contended that the asymmetry is robust and important for efforts aimed at modelling when a saccade is initiated as a function of ensuing saccade direction.

## Introduction

*“…the model and good data go hand in hand in advancing the field.” * (p. 7, 1)

The visual field is the area of visibility, with respect to the current eye fixation. An object positioned above or below the current eye fixation is respectively in the current upper or lower visual field. It is well established that temporal processing along the vertical

meridian of the visual field is asymmetric (2, 3, 4, 5). For example, key-press/manual reactions (MRTS) are usually faster for targets that appear in the lower, not upper visual field. These

behavioral findings are supported by physiological findings in the forms of shorter latency, and larger amplitude evoked responses in retinal and occipital areas (see also 3 for a review, 6, 7, 8). The accepted explanation is that the lower visual field advantage for manual reactions is a result of an asymmetrical representation of the lower visual field in the (upper) retina and (superior) visual cortex areas (see 3, 9, 10).

Given the structural limitations of the visual system, only a small central area of the visual field is accessible to detailed scrutiny. As such, it is necessary to make fast eye movements (i.e., saccades) to bring peripheral areas into central processing range. Saccades continually change the position of the visual field. In contrast to the lower visual field advantage for manual responses, the latency to initiate a saccade towards a target is usually shorter by 20ms- 50ms, if the target appears in the upper visual field (e.g., 11, see also 12 for a mini review). This upper visual field advantage for saccade reaction times (SRTs) is well established in the literature (11, 13, 14, 15, 16, 17, 18, 19, 20, 21, 22, 23). As with MRTs, asymmetry in SRTs are also supported by physiological findings of sharper, faster and stronger upper visual field representation in subcortical and cortical oculomotor areas (21, 24, 25). The characteristics of SRTs are important in clinical vision sciences. For example, they have a diagnostic value in some movement disorders (e.g., Huntington’s disease, and recessive cerebellar ataxia; see 26, 27, 28). Hence, typical SRT asymmetries may provide insight on the functioning of a healthy brain.

While knowledge of SRT characteristics is useful (e.g., in clinical vision sciences), there is a limitation with respect to temporal characteristics of saccadic *exploration *of targets in a visual field. In SRT tasks, the goal is to prepare and initiate a saccade as quickly as possible to one of a number of preset alternative locations. This is not the case during typical exploration of a visual scene, where the viewer decides autonomously, where to direct saccades. A comprehensive understanding of the visual brain (leading to an ability to predict, model, and clinically manage its operations) requires research on saccadic exploration characteristics. Characteristics may be classified as *when* a saccade is initiated, and *where* the saccade goes (i.e., directional and distance control). Many computational models of saccade exploration have focused on where saccades go in the visual field (e.g., 29, 30, 31, 32, 33). Towards real-time modelling of saccadic exploration (e.g., 34, 35, 36, 37), it is necessary to determine *when* a saccade is initiated in association with *where* the saccade goes in the visual field (e.g., 12, 38, 39, 40). In the context of saccadic exploration, a saccade is initiated after the duration of an eye fixation. Following three recent studies (12, 38, 39), we refer to this duration as the presaccadic fixation duration (PSFD). Unlike SRTs, PSFDs are confounded by the time taken to exploit information in the area fixated and the time required to program a saccade for exploration (41, 42). Un-confounding the two variables is beyond the goal of the present meta-analysis.

It has been reported that a vertical visual field asymmetry for PSFDs exists, such that fixation durations are shorter before the eyes are directed above eye fixation (12, 38, 39). A need exists to determine the replicability and generality of this asymmetry if it is to be considered in real-time modelling of saccadic exploration (see 43, 44 for discussions of the replication crisis in science). In the present study we report a meta-analysis of published and unpublished results of the vertical asymmetry in PSFDs. The goal was to summarize results of a diverse set of saccadic exploration studies, and to quantify the extent of visual field asymmetry for PSFD in these studies. If the purported asymmetry in PSFDs is reliable (12, 38, 39), a significant asymmetry was expected in overall effect size across different datasets. In addition to determining the overall effect size, four potential moderators of the effect were considered. First we reasoned that the differences in demands between searching for a target and viewing a scene justifies the inclusion of Task (Visual Search vs Scene Viewing) as a moderator variable. Second, towards safeguarding against publication bias, unpublished data were included in the meta-analysis. For this reason, the variable Source (Published vs Unpublished) was included as a moderator. Some of the datasets included in the meta-analysis were created specifically to test for the existence of the purported asymmetry in PSFDs. To assess the potential influence of verification bias, Pre-planned Test (Yes vs No) was included as a moderator. Finally, some studies artificially constrained the observer’s head in a chinrest. Towards ecological validity, it was useful to examine the constraint of a chinrest as moderator: Chinrest (Yes vs No).

## Method

### Data Selection Criteria

Eye movement data were included from research conducted at three laboratories. As our concern was saccadic behavior, the specific aims of the different studies considered were not important. Published and new datasets were considered, to safeguard against publication bias. We were particularly interested in situations that reasonably simulated realistic looking demands. For inclusion in the meta-analysis, saccadic explorations had to have been made without gaze-contingent (GC) visual field restrictions on complex visual displays. Our inclusion criteria allowed real-world scenes, fractals, roadmaps, picture webpages, random dot displays, and ambiguous inkblot images. Studies that utilized structured alpha-numeric characters were excluded. For studies that utilized some GC manipulations, only the conditions without GC manipulations were included.

### Description of Selected Datasets

**Figure 1.**
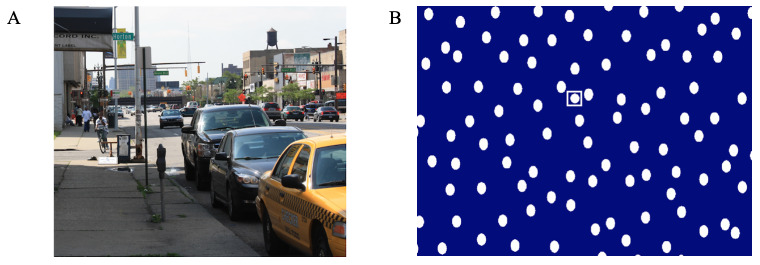
An urban scene used in a scene viewing task, and a sample display with a target circle among ovals, used in visual search tasks included in the new eye movement data for the meta-analysis. The square in the visual search display depicts for the reader, the position of the target in this particular display.

Six of the datasets were from 3 published studies that reported the vertical asymmetry in PSFDs during saccadic exploration (12, 38, 39). Eight of the datasets were from 3 published studies of saccadic exploration that had not previously checked for the vertical asymmetry in PSFDs (45, 46, 47. Nine new eye-movement datasets were from the laboratories of the authors, and were included in the analysis, to minimize publication bias. Table 1 presents brief descriptions of the methods utilized for the datasets included in the meta-analysis. Figure 1 shows one urban scene used in previously unpublished scene viewing tasks, and a sample trial display with a target circle among ovals, used in visual search tasks. In one scene-viewing task, the stimuli were randomly chosen webpages, containing pictures and words. All other stimulus sets are well described in the published datasets (45, 46, 47), and briefly described in Table 1.

In sum, 23 datasets were included in the meta-analysis (14 visual search, and 9 scene-viewing datasets). Eye movements were tracked using Eyelink systems at 250Hz, or higher, as participants explored presentations on a computer screen. All data collection was approved by the relevant university Institutional Review Boards. They were conducted in accordance with the Belmont Report, and the Code of Ethics of the World Medical Association (Declaration of Helsinki). Participants were healthy adults with normal, or corrected-to-normal visual acuity.

### Data Analysis

In-house codes were used to extract PSFDs associated with ensuing saccade directions within the quadrants of the visual field (e.g., 38, 39). A priori paired samples t tests were conducted to test for significant differences between PSFDs for saccades directed within the 90 deg radius above, and below each current eye fixation. The meta-analysis was conducted using Comprehensive Meta-Analysis V3 software. The data entered were the mean PSFDs for saccades directed within the 90 deg wedge above and below current eye fixation, the sample size, and the paired samples t value. Effect sizes for the difference in PSFDs when saccades were directed into the upper visual field (UpVF) and lower visual field (LoVF) were quantified as Hedge’s g (48). Heterogeneity among datasets was assessed as the percentage of variance in effect (i.e., *I² %* *= 100(Q - df)/Q*; (49), where in the present context, Q is the result of Cochran’s $\chi^{2}$ test with *df* degrees of freedom).

## Results

**Figure 2.**
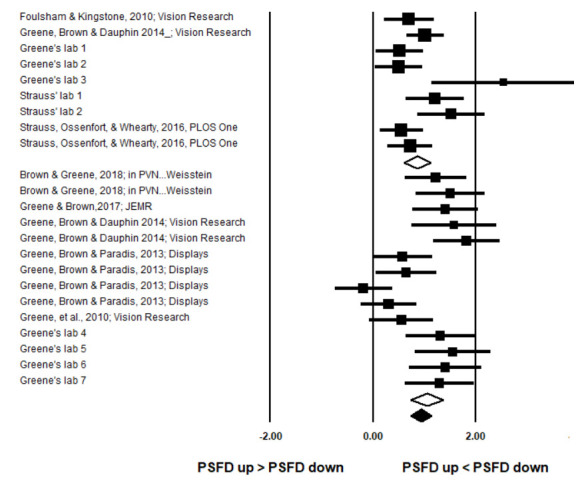
Standardized mean difference (as Hedge’s g) between PSFDs for up-directed and down-directed saccades. The sequence of datasets in the forest plot is the same as the sequence presented in Table 1. Error bars represent 95% confidence intervals. The sizes of the black squares reflect sample sizes. Pooled effect sizes are shown as diamonds for scene viewing and visual search (open), and overall (closed). The horizontal extent of the diamonds depict 95% confidence intervals. The pooled effect sizes indicate an asymmetry such that PSFDs are shorter for up-directed than down-directed saccades.

In Table 1, we have presented PSFDs for paired samples comparisons of mean PSFDs associated with UpVF-directed and LoVF-directed saccades. A priori paired samples t tests revealed significant vertical visual field asymmetries in 19 of the 23 datasets (p < .05). The table shows that overall, there was an asymmetry in PSFDs such that PSFDs were shorter by 25ms for up-directed saccades. Standardized extent of asymmetry in PSFDs was quantified by Hedge’s g for each dataset. There was statistically significant heterogeneity among the 23 datasets (*Q*(22) = 68.16, p < .01; percentage of variance in effect sizes due to heterogeneity: *I² %* = 67.72%). Given the heterogeneity of variances, a random effects model was preferred over a fixed effect model to quantify the pooled effect size. The random effects model produces a wider confidence interval, to compensate for the heterogeneity. The pooled effect size (shown as a diamond at the bottom of Figure 2) indicated significant asymmetry such that during saccadic exploration in a variety of tasks, PSFDs for saccades into the upper visual field were typically shorter than PSFDs for saccades into the lower visual field (pooled g overall = 0.97, z= 9.09, p < .01).

### Moderator: Task (Visual Search vs Scene Viewing) 

Analysis of data subsets showed statistically significant heterogeneity among Scene Viewing tasks (*Q*(8) = 19.07, p < .01; *I² %* = 58.05%), and Visual Search tasks (*Q*(13) = 47.18, p < .01; *I² %* = 72.44%). With a random effects model, there was a significant asymmetry such that during saccadic exploration in Visual Search and in Scene Viewing tasks, PSFDs for saccades into the upper visual field were typically shorter than PSFDs for saccades into the lower visual field (pooled g for Visual Search = 1.05, z = 6.34; pooled g for scene viewing = .86, z = 6.56, all ps < .01).

The variable Task(Visual Search vs Scene Viewing), was not significant as a moderator *Q*(1) = .76, p > .05 (see also, overlapping 95% confidence interval diamonds for pooled Visual Search and pooled Scene Viewing effects in Figure 2). In sum, the effect size was not different between Visual Search and Scene Viewing tasks examined in the meta-analysis.

### Moderator: Source (Published vs Unpublished)

Analysis of data subsets showed statistically significant heterogeneity among Published studies (*Q*(13) = 42.92, p < .01; *I² %* = 69.71%), and Unpublished studies (*Q*(8) = 21.71, p < .01; *I² %* = 63.15%). With a random effects model, there was a significant asymmetry such that during saccadic exploration in Published and Unpublished datasets, PSFDs for saccades into the upper visual field were typically shorter than PSFDs for saccades into the lower visual field (pooled g for Published studies = .85, z = 6.37; pooled g for Unpublished studies = 1.19, z = 6.65, all ps < .01).

The variable Source (Published vs Unpublished), was not significant as a moderator *Q*(1) = 2.23, p > .05. In sum, the effect size was not different between Published and Unpublished studies examined in the meta-analysis.

### Moderator: Pre-planned Test (Yes vs No)

Only visual search studies were in the pool of studies designed specifically to test for the purported asymmetry in PSFDs. There was no significant heterogeneity among these studies (i.e., Yes studies; *Q*(6) = 2.15, p > .05; *I² %* = 0%). In contrast, there was significant heterogeneity among (visual search and scene-viewing) studies that were not designed specifically to test for the purported asymmetry (i.e., No studies; *Q*(15) = 40.58, p < .01; *I² %* = 63.03%).

With a random effects model, there was a significant asymmetry such that during saccadic exploration in Yes, and No studies, PSFDs for saccades into the upper visual field were typically shorter than PSFDs for saccades into the lower visual field (pooled g for Yes studies = 1.46, z = 11.45; pooled g for No studies = .77, z = 6.79, all ps < .01).

The variable Pre-planned Test (Yes vs No), was a significant moderator *Q*(1) = 15.97, p < .01. In sum, the effect size was larger for studies (limited to visual search studies in our meta-analysis) specifically designed to test for the purported asymmetry in PSFDs.

### Moderator: Chinrest (Yes vs No)

Analysis of data subsets showed statistically significant heterogeneity among studies that used a chinrest (*Q*(9) = 31.55, p < .01; *I² %* = 71.47%), and studies that did not (*Q*(12) = 31.44, p < .01; *I² %* = 61.84%). With a random effects model, there was a significant asymmetry such that during saccadic exploration in Chinrest and No Chinrest datasets, PSFDs for saccades into the upper visual field were typically shorter than PSFDs for saccades into the lower visual field (pooled g for chinrest studies = .79, z = 4.87; pooled g for no chinrest studies = 1.13, z = 8.09, all ps < .01).

The variable Chinrest (Yes vs No), was not significant as a moderator *Q*(1) = 2.43, p > .05. In sum, the effect size was not different between Chinrest and No Chinrest studies examined in the meta-analysis.

## Discussion

An asymmetry has been reported for PSFDs associated with saccades directed within the vertical visual field (12, 39). An open question is the robustness of this reported asymmetry for future research endeavors. The present meta-analytic study, the first of its kind, had two objectives: to report PSFDs for vertically-directed saccades in a diverse set of saccadic exploration studies, and to quantify the extent of vertical asymmetry in PSFDs. Overall, there was a statistically significant asymmetry in PSFDs such that during saccadic exploration, fixation durations were briefer if saccades were directed into the upper visual field. The results of the meta-analysis support the published reports of PSFD asymmetry. The effect was not moderated by saccade exploration task (i.e., Visual Search vs Scene Viewing), publication bias, or the constraint of a chinrest. Whether or not a study was designed specifically to test for the PSFD effect did matter, such that studies designed to test for the effect showed a larger effect size. However, all the studies designed specifically to test for the effect were visual search studies. In the future, it would be useful to conduct a planned comparison of the effect for scene-viewing and visual search of the same stimulus set.

### Speculative explanations 

Movement of sensors relative to objects in the environment is important for learning regularities of our environment (e.g. Hawkins, Ahmad, and Cui, 2017). As the visual brain reaches out to engage with the world, information above or below the current eye fixation is respectively positioned in the current upper or lower field, and saccades continually change what constitutes the upper and lower visual field. To date, it has not been established why PSFDs tend to be shorter when the ensuing saccade is directed upwards. An account is summarized here, to stimulate future research efforts on the question.

If one considers global optic flow while walking and looking straight ahead, the speed/velocity across the retina is least at the fixation point, and increasing in the periphery.  In an outdoor setting with sky above, a greater amount of such stimulation would be in the lower visual field. A “check and detect” behavior pattern stemming from ecological constraints (e.g., 2) may underlie the vertical visual field asymmetry for PSFDs. Given the position of the eyes atop the human body, information in far/extra-personal space tends to be in the UpVF, and information near the body tends to be in the LoVF. Hence, as one moves within one’s environment, gaze is typically directed some distance ahead to search for, and detect new information in the slower flowing upper optic array (i.e. UpVF). As one is covertly aware of near-body/peripersonal and ground-level space in the LoVF, one rarely looks down to check already covertly attended things in one’s faster flowing ground level. Occasionally, one makes saccades downwards to check the information in the current LoVF. Support for a “check and detect” behavior pattern comes from laboratory findings that saccades are more likely to be directed upwards to discern information in the current UpVF (e.g., 12) (see also 50 for possible influences of perceived scene orientation). The vertical asymmetry in PSFDs elicited by a “check and detect” mechanism may be grounded in neuroscience.

From a visibility perspective, greater representation of the lower visual field in the (upper) retina and (superior) visual cortex areas (see 3, 9, 10) results in higher contrast sensitivity below eye fixation. Hence, there may be a greater urgency to terminate a fixation, and make a saccade upwards, because for a given radius, objects above fixation are seen less clearly during a fixation. The visibility perspective predicts shorter fixation durations for ensuing saccades directed upwards because there is a greater need to disambiguate by looking, objects above fixation.

Beyond a visibility perspective, magnetoencephalography results (21) suggest that frontal cortical areas involved in saccade preparation respond earlier when saccades are directed upwards, than when saccades are directed downwards. Additionally, when the intention is to make a saccade upwards, frontal cortex activity is lower than when a downward saccade is intended (21). The findings suggest that saccade programming may require less cortical effort for saccades directed upwards in the visual field. In turn, less cortical effort may encourage shorter fixation durations for ensuing saccades directed upwards.

Beyond cortical effort, saccades are controlled by the cortex through the sub-cortical superior colliculus (see Goldberg and Walker, 2013, for an introduction). Recent findings suggest that the representation of the vertical visual field is not uniform in the superior colliculus (Hafed & Chen, 2016). Specifically, the UpVF representation in the superior colliculus is faster than the LoVF representation (Hafed & Chen, 2016). Indeed, the asymmetrical representation of the vertical visual field by the superior colliculus may also contribute to the asymmetry in vertical PSFDs. Together, a combination of adaptive mechanisms (e.g. check and detect behavior), and retinal, cortical and subcortical mechanisms may reasonably underlie the observed shorter fixation durations before saccades are directed upwards in the visual field. Perhaps, future animal neuroscience experiments could manipulate aspects of the saccadic circuitry to test the speculations proposed.

### A word on SRTs and PSFDs

Whereas effect sizes in our meta-analysis have demonstrated the robustness of the vertical field asymmetry in PSFDs (irrespective of statistical significance in null hypothesis tests), it is informative to ask whether SRTs are also similarly robust. Greene, Brown and Dauphin (2014) have argued that while the high level of experimental control in SRT tasks is useful for determining the operations of the saccadic circuitry, a limitation is that the task demand is different from saccadic exploration. Reasonably, SRTs index the time required to program a saccade, based on exogenously oriented attention. In contrast, during saccadic exploration (e.g., visual search and scene viewing), observers typically direct their attention endogenously, such that PSFDs heavily reflect both the time required to program a saccade *and* the amount of time taken to process the currently-fixated element in the visual field (see also, 36, 37). The systematic asymmetry in PSFDs probably reflects this added saccade programming time. To date, no meta-analysis has been conducted to ascertain this. Despite the difference in focus between SRTs and PSFDs, in their overview of the literature, Greene, Brown and Dauphin (2014) have suggested that the SRT asymmetry is noteworthy for convergent validity and computational modelling of saccadic behavior.

In the present study, the advantage of a meta-analysis is that it quantifies an effect in a manner that does not rely on a single dataset. Although selected datasets were not all created using the same methodology, the underlying mechanism of interest was the same--that is to say, saccadic explorations were made without GC manipulations on complex visual displays. The present study indicates that when there are no GC spatial constraints, there is a robust PSFD asymmetry for up- and down-directed saccades. Despite the difficulty of separating exploitation time from saccade preparation time in saccadic exploration tasks, PSFDs, like SRTs, are systematically shorter when the ensuing saccade is directed into the UpVF. Given the similarity in characteristics between SRTs and PSFDs, we contend that low-level saccade programming mechanisms affect PSFDs in predictable ways, which make PSFDs relevant in clinical vision sciences where they may have a diagnostic value in some movement disorders (e.g., Huntington’s disease, and recessive cerebellar ataxia; see 26, 27, 28). It has also recently come to our attention that there are stable individual differences in saccadic indices (51, 52, 53). This makes the reported asymmetry a worthwhile avenue for research on psychometric dynamics. Finally, we contend that the asymmetry in PSFDs is important for efforts aimed at modelling *when* a saccade is initiated as a function of ensuing saccade direction in the visual field (e.g., 34, 35, 37, 54).

### Limitations

A potential limitation of the presented meta-analysis is the small number of datasets considered. Three moderators were not statistically significant. However, without regard to statistical significance, moderator analyses indicated a slightly larger asymmetry for visual search than scene viewing tasks (Δg= 1.05 - .86 = .19), unpublished than published studies (Δg = 1.19 - .85 = .34), and No Chinrest than Chinrest studies (Δg = 1.13 - .79 = .34). Perhaps most interesting from a theoretical point of view is the potential larger effect when a chin rest is not utilized. An ecological account of the asymmetry in vertical PSFDs predicts that the effect should be hindered by artificial head constraints. The availability of a larger set of studies in the future may contribute to assessing statistical significance of artificial (chinrest) constraints in the size of the effect.

### Conclusion

We have shown that vertical visual field asymmetry for PSFDs is robust. Despite the small number of studies considered, it may reasonably be argued that the benefits of the present meta-analysis far outweigh its limitation. Of course, meta-analyses of the present sort may be facilitated by the deposition of data in publicly accessible databases (e.g. Dryad data repository, https://datadryad.org/). Ideally, for each fixation, deposited data should have at least, the components presented in italics, below.

*<sampling rate>*

*<eye recorded>*

*<fixation_start_time_stamp> *(optional)

*<fixation_end_time_stamp> *(optional)

*<fixation_duration_in ms>*

*<average_horizontal_eye_position>*

*<average_vertical_eye_position>*

To conclude, we predict that the vertical visual field asymmetry is present in datasets from other laboratories and encourage groups of researchers to conduct similar meta-analyses, towards establishing trustworthiness in the kinds of data used to guide computational modelling of real-time saccadic exploration. Indeed, “… the model and good data go hand in hand in advancing the field.” (p. 7, 1). Given the theory of an ecology-driven functional specialization of the visual field above and below eye fixation (Previc, 1990), it may reasonably be predicted that the asymmetry in PSFDs is most apparent under typical environmental demands.

**Table 1:** Description of tasks included in the meta-analysis. Presaccadic Fixation Durations (PSFDs) for up-directed and down-directed saccades, and effect sizes (Hedge’s g) for paired samples comparisons.

Study	Condition & stimuli in study	Sample size	PSFD up-saccades (ms)	PSFD down-saccade (ms)	Difference in means (ms)	Hedge’s g	Std Err
Foulsham & Kingstone (2010)	2-second viewing of natural scenes and fractals (encoding phase)	20	243.55	269.17	25.62	0.702	0.242
Greene et al., (2014)	Engaged viewing of ambiguous (Rorschach) inkblots.	44	327.28	355.25	27.97	1.015	0.183
Greene’s lab ~	15-second viewing to rate the attractiveness of 9 urban scenes.	20	252.40	270.71	18.31	0.515	0.230
Greene’s lab ~	15-second viewing to rate the secureness of 9 urban scenes.	19	252.98	278.63	25.65	0.499	0.234
Greene’s lab ~	Engaged viewing of university webpages.	8	185.91	227.49	41.58	2.539	0.708
Strauss’ lab ~	Passive viewing of IAPS unpleasant scenes.	20	272.89	300.2	27.31	1.204	0.287
Strauss’ lab ~	Passive viewing of IAPS unpleasant scenes.	19	265.02	302.36	37.34	1.527	0.331
Strauss et al., (2016)	5-second viewing of unpleasant IAPS scenes, while distracted by thoughts of unrelated neutral objects.	25	291.77	314.82	23.05	0.552	0.209
Strauss et al., (2016)	5-second viewing of unpleasant IAPS scenes, while reappraising them to be neutral.	25	282.27	304.66	22.39	0.726	0.219
Brown & Greene, (2018)	Visual search for low contrast square in a random gray-dot display.	18	268.08	297.77	29.69	1.218	0.303
Brown & Greene, (2018)	Visual search for low contrast square in a random red-dot display.	18	280.39	313.86	33.47	1.506	0.337
Greene & Brown, (2017)	Visual search for low contrast square in a random gray-dot display.	18	256.14	278.71	22.57	1.409	0.325
Greene et al., (2014)	Visual search for low contrast checkerboard in a random gray-dot display.	12	213.84	255.27	41.43	1.579	0.420
Greene et al., (2014)	Visual search for low contrast square in a random gray-dot display.	24	243.18	286.52	43.34	1.822	0.329
Greene et al., (2013)	Monocular visual search for high contrast square in a random gray-dot display.	12	218.29	227.82	9.53 ^#^	0.577	0.293
Greene et al., (2013)	Monocular visual search for low contrast square in a random gray-dot display.	12	341.6	366	24.40	0.647	0.299
Greene et al., (2013)	Visual search for high contrast square in a random gray-dot display.	11	206.92	201.69	-5.23 ^ # ^	-0.186	0.281
Greene et al., (2013)	Visual search for low contrast square in a random gray-dot display.	12	305.56	317.6	12.04 ^ # ^	0.306	0.276
Greene et al., (2010)	Visual search of roadmaps.	10	242.13	257.91	15.78 ^ # ^	0.552	0.314
Greene’s lab ~	3-second visual search-white circles on blue background.	15	201.52	220.41	18.89	1.313	0.342
Greene’s lab ~	3-second visual search-white circles on green background.	15	198.24	224.16	25.92	1.553	0.374
Greene’s lab ~	3-second visual search-white circles on red background.	15	200.31	220.96	20.65	1.411	0.355
Greene’s lab ~	Visual search for low contrast square in a random gray-dot display.	15	206.02	234.46	28.44	1.296	0.340
							
Means			247.07	272.25	25.18		

*Notes.* ^~ ^New Data. ^#^Non-significant paired samples t IAPS: International Affective Picture System https://csea.phhp.ufl.edu/media/iapsmessage.html

## Ethics and Conflict of Interest

The author(s) declare(s) that the contents of the article are in agreement with the ethics described in http://biblio.unibe.ch/portale/elibrary/BOP/jemr/ethics.html and that there is no conflict of interest regarding the publication of this paper. 

## Acknowledgements

We appreciate the recommendations of Jessica Matchynski-Franks and Latoya Taylor in the planning of the meta-analysis. We acknowledge Claudia Bernasconi and Alan Hoback for providing urban scenes. The comments of all formal, and informal reviewers of this work are greatly appreciated. Correspondence should be addressed to H. Greene, Department of Psychology, University of Detroit Mercy, Detroit, MI, 48221 (email: greenehh@udmercy.edu).

## References

[b11] Abegg, M. , Pianezzi, D. , & Barton, J. ( 2015). A vertical asymmetry in saccades. Journal of Eye Movement Research, 8( 5), 1–10. 10.16910/jemr.8.5.3 1995-8692

[b13] Bell, A. H. , Everling, S. , & Munoz, D. P. ( 2000). Influence of stimulus eccentricity and direction on characteristics of pro- and antisaccades in non-human primates. Journal of Neurophysiology, 84( 5), 2595–2604. 10.1152/jn.2000.84.5.2595 0022-3077 11068001

[b38] Brown, J. M. , & Greene, H. H. ( 2018). We’re going to study the mind In J. M. Brown ( Ed.), Pioneer Visual Neuroscience: A Festschrift for Naomi Weisstein (pp. 6–32). Routledge 10.4324/9781315170183-2

[b51] Castelhano, M. S. , & Henderson, J. M. (2008).The influence of color on the perception of scene gist. Journal of Experimental Psychology. Human Perception and Performance,34(3),660–675. 10.1037/0096-1523.34.3.660 0096-1523 18505330

[b24] Dräger, U. C. , & Hubel, D. H. (1976).Topography of visual and somatosensory projections to mouse superior colliculus. Journal of Neurophysiology,39(1),91–101. 10.1152/jn.1976.39.1.91 0022-3077 1249606

[b50] Foulsham, T. , & Kingstone,A. (2010).Asymmetries in the direction of saccades during perception of scenes and fractals: Effects of image type and image features. Vision Research,50(8),779–795. 10.1016/j.visres.2010.01.019 0042-6989 20144645

[b52] Foulsham,T. , Frost,E. , & Sage,L. (2018).Stable individual differences predict eye movements to the left, but not handedness or line bisection. Vision Research,144,38–46. 10.1016/j.visres.2018.02.002 0042-6989 29499212

[b14] Goldring,J. , & Fischer,B. (1997).Reaction times of vertical prosaccades and antisaccades in gap and overlap tasks. Experimental Brain Research,113(1),88–103. 10.1007/BF02454145 0014-4819 9028778

[b46] Greene,H. H. , Pollatsek,A. , Masserang,K. , Lee,Y. J. , & Rayner,K. (2010).Directional processing within the perceptual span during visual target localization. Vision Research,50(13),1274–1282. 10.1016/j.visres.2010.04.012 0042-6989 20399222 PMC2878909

[b45] Greene,H. H. , Brown,J. M. , & Paradis,B. A. (2013).Luminance contrast and the visual span during visual target localization. Displays,34(1),27–32. 10.1016/j.displa.2012.11.005 0141-9382

[b12] Greene,H. H. , Brown,J. M. , & Dauphin,B. (2014).When do you look where you look? A visual field asymmetry. Vision Research,102(0),33–40. 10.1016/j.visres.2014.07.012 0042-6989 25094053

[b39] Greene,H. H. , & Brown,J. M. (2017).Where did I come from? Where am I going? Functional differences in visual search fixation duration. Journal of Eye Movement Research,10(1),1–13. 10.16910/jemr.10.1.5 1995-8692 PMC7141091 33828646

[b15] Hackman,R. B. (1940).An experimental study of variability in ocular latency. Journal of Experimental Psychology,27(5),546–558. 10.1037/h0053943 0022-1015

[b25] Hafed,Z. M. , & Chen,C.-Y. (2016).Sharper, stronger, faster upper visual field representation in primate superior colliculus. Current Biology,26(13),1647–1658. 10.1016/j.cub.2016.04.059 0960-9822 27291052

[b6] Hagler DJ,Jr (2014).Visual field asymmetries in visual evoked responses. Journal of Vision,14(14), 10.1167/14.14.13. PMC4274841 25527151

[b48] Hedges L , Olkin I. (1985).Statistical Methods in Meta-Analysis San Diego: Academic Press

[b53] Henderson,J. M. , & Luke,S. G. (2014).Stable individual differences in saccadic eye movements during reading, pseudoreading, scene viewing, and scene search. Journal of Experimental Psychology. Human Perception and Performance,40(4),1390–1400. 10.1037/a0036330 0096-1523 24730735

[b16] Heywood,S. , & Churcher,J. (1980).Structure of the visual array and saccadic latency: Implications for oculomotor control. The Quarterly Journal of Experimental Psychology,32(2),335–341. 10.1080/14640748008401169 0033-555X 7433625

[b49] Higgins,J. P. T. , & Thompson,S. G. (2002).Quantifying heterogeneity in a meta-analysis. Statistics in Medicine,21(11),1539–1558. 10.1002/sim.1186 0277-6715 12111919

[b9] Holmes,G. M. (1945).Ferrier Lecture - The organization of the visual cortex in man. Proceedings of the Royal Society of London. Series B, Biological Sciences,132(869),348– 361. 10.1098/rspb.1945.0002 0080-4649

[b17] Honda,H. , & Findlay,J. M. (1992).Saccades to targets in three-dimensional space: Dependence of saccadic latency on target location. Perception & Psychophysics,52(2),167–174. 10.3758/bf03206770 10.3758/bf03206770 0031-5117 1508624

[b29] Itti,L. , & Koch,C. (2000).A saliency-based search mechanism for overt and covert shifts of visual attention. Vision Research,40(10-12),1489–1506. 10.1016/S0042-6989(99)00163-7 0042-6989 10788654

[b7] Kremlácek,J. , Kuba,M. , Chlubnová,J. , & Kubová,Z. (2004).Effect of stimulus localisation on motion-onset VEP. Vision Research,44(26),2989– 3000. 10.1016/j.visres.2004.07.002 0042-6989 15474572

[b26] Lasker,A. G. , & Zee,D. S. (1997).Ocular motor abnormalities in Huntington’s disease. Vision Research,37(24),3639–3645. 10.1016/S0042-6989(96)00169-1 0042-6989 9425536

[b34] Laubrock,J. , Cajar,A. , & Engbert,R. (2013).Control of fixation duration during scene viewing by interaction of foveal and peripheral processing. Journal of Vision (Charlottesville, Va.),13(12),11.Advance online publication. 10.1167/13.12.11 1534-7362 24133291

[b43] Maxwell,S. E. , Lau,M. Y. , & Howard,G. S. (2015).Is psychology suffering from a replication crisis? What does “failure to replicate” really mean? The American Psychologist,70(6),487–498. 10.1037/a0039400 0003-066X 26348332

[b18] Miles,W. R. (1936).The reaction time of the eye. Psychological Monographs,47(2),268–293. 10.1037/h0093418 0096-9753

[b30] Najemnik,J. , & Geisler,W. S. (2009).Simple summation rule for optimal fixation selection in visual search. Vision Research,49(10),1286–1294. 10.1016/j.visres.2008.12.005 0042-6989 19138697

[b36] Nuthmann,A. , & Henderson,J. M. (2010).Object-based attentional selection in scene viewing. Journal of Vision (Charlottesville, Va.),10(8),20.Advance online publication. 10.1167/10.8.20 1534-7362 20884595

[b35] Nuthmann,A. (2017).Fixation durations in scene viewing: Modeling the effects of local image features, oculomotor parameters, and task. Psychonomic Bulletin & Review,24(2),370–392. 10.3758/s13423-016-1124-4 1069-9384 27480268 PMC5390002

[b54] Nuthmann,A. , Smith,T. J. , Engbert,R. , & Henderson,J. M. (2010).CRISP: A computational model of fixation durations in scene viewing. Psychological Review,117(2),382–405. 10.1037/a0018924 0033-295X 20438231

[b31] Parkhurst,D. , Law,K. , & Niebur,E. (2002).Modeling the role of salience in the allocation of overt visual attention. Vision Research,42(1),107–123. 10.1016/S0042-6989(01)00250-4 0042-6989 11804636

[b27] Patel,S. S. , Jankovic,J. , Hood,A. J. , Jeter,C. B. , & Sereno,A. B. (2012).Reflexive and volitional saccades: Biomarkers of Huntington disease severity and progression. Journal of the Neurological Sciences,313(1-2),35–41. 10.1016/j.jns.2011.09.035 0022-510X 22018763 PMC3254798

[b19] Pitzalis,S. , & Di Russo,F. (2001).Spatial anisotropy of saccadic latency in normal subjects and brain-damaged patients. Cortex,37(4),475–492. 10.1016/s0010-9452(08)70588-4 10.1016/s0010-9452(08)70588-4 0010-9452 11721860

[b8] Portin,K. , Vanni,S. , Virsu,V. , & Hari,R. (1999).Stronger occipital cortical activation to lower than upper visual field stimuli. Neuromagnetic recordings. Experimental Brain Research,124(3),287– 294. 10.1007/s002210050625 0014-4819 9989434

[b2] Previc,F. H. (1990).Functional specialization in the lower and upper visual fields in humans: Its ecological origins and neurophysiological implications. Behavioral and Brain Sciences,13(3),519– 575. 10.1017/S0140525X00080018 0140-525X

[b32] Rao,R. P. N. , Zelinsky,G. J. , Hayhoe,M. M. , & Ballard,D. H. (2002).Eye movements in iconic visual search. Vision Research,42(11),1447–1463. 10.1016/S0042-6989(02)00040-8 0042-6989 12044751

[b41] Rayner,K. (1998).Eye movements in reading and information processing: 20 years of research. Psychological Bulletin,124(3),372–422. 10.1037/0033-2909.124.3.372 0033-2909 9849112

[b1] Rayner,K. (2009).Eye movements and attention in reading, scene perception, and visual search. Quarterly Journal of Experimental Psychology,62(8),1457– 1506. 10.1080/17470210902816461 1747-0218 19449261

[b20] Schlykowa,L. , Hoffmann,K.-P. , Bremmer,F. , Thiele,A. , & Ehrenstein,W. H. (1996).Monkey saccadic latency and pursuit velocity show a preference for upward directions of target motion. An International Journal for the Rapid Communication of Research in Neuroscience.,7(2),409–412. 10.1097/00001756-199601310-00008 0959-4965 8730793

[b42] Sheth,B. R. , & Young,R. (2016).Two Visual Pathways in Primates Based on Sampling of Space: Exploitation and Exploration of Visual Information. Frontiers in Integrative Neuroscience,10(37),37.Advance online publication. 10.3389/fnint.2016.00037 1662-5145 27920670 PMC5118626

[b44] Shrout,P. E. , Rodgers,J. (2018).Psychology, Science, and Knowledge Construction: Broadening Perspectives from the Replication Crisis.487–510. 10.1146/annurev-psych-122216-011845 29300688

[b10] Silva,M. F. , Brascamp,J. W. , Ferreira,S. , Castelo-Branco,M. , Dumoulin,S. O. , & Harvey,B. M. (2018).Radial asymmetries in population receptive field size and cortical magnification factor in early visual cortex. NeuroImage,167,41–52. 10.1016/j.neuroimage.2017.11.021 1053-8119 29155078

[b3] Skrandies W. (1987).The upper and lower visual field of man: Electrophysiological and functional differences. Progress in sensory physiology. 8 2– 93

[b47] Strauss,G. P. , Ossenfort,K. L. , & Whearty,K. M. (2016).Reappraisal and Distraction Emotion Regulation Strategies Are Associated with Distinct Patterns of Visual Attention and Differing Levels of Cognitive Demand. PLoS One,11(11),e0162290. 10.1371/journal.pone.0162290 1932-6203 27855175 PMC5113864

[b40] Tatler,B. W. , & Vincent,B. T. (2009).The prominence of behavioural biases in eye guidance. Visual Cognition,17(6-7),1029–1054. 10.1080/13506280902764539 1350-6285

[b28] Termsarasab,P. , Thammongkolchai,T. , Rucker,J. C. , & Frucht,S. J. (2015).The diagnostic value of saccades in movement disorder patients: A practical guide and review. Journal of Clinical Movement Disorders,2(1),14. 10.1186/s40734-015-0025-4 2054-7072 26788350 PMC4710978

[b37] Trukenbrod,H. A. , & Engbert,R. (2014).ICAT: A computational model for the adaptive control of fixation durations. Psychonomic Bulletin & Review,21,907–934. 10.3758/s13423-013-0575-0 1069-9384 24470305

[b23] Tzelepi,A. , Yang,Q. , & Kapoula,Z. (2005). The effect of transcranial magnetic stimulation on the latencies of vertical saccades 67–77. 10.1007/s00221-005-2250-9 15915351

[b21] Tzelepi,A. , Laskaris,N. , Amditis,A. , & Kapoula,Z. (2010).Cortical activity preceding vertical saccades: A MEG study. Brain Research,1321(0),105–116. 10.1016/j.brainres.2010.01.002 0006-8993 20079341

[b4] Woodworth,R. S. (1938).Experimental psychology.H. Holt and Company.

[b33] Zelinsky,G. J. (2008).A theory of eye movements during target acquisition. Psychological Review,115(4),787–835. 10.1037/a0013118 0033-295X 18954205 PMC2577318

[b22] Zhou,W. , & King,W. M. (2002).Attentional sensitivity and asymmetries of vertical saccade generation in monkey. Vision Research,42(6),771–779. 10.1016/S0042-6989(01)00319-4 0042-6989 11888542

[b5] Zhou,Y. , Yu,G. , Xuefei,Y. , Wu,S. , & Zhang,M. (2017).Asymmetric representations of upper and lower visual fields in egocentric and allocentric references. 10.1167/17.1.9 28114481

